# Molecular discernment and histopathological features of oncogenic Marek’s disease virus among different farmed avian species in Egypt

**DOI:** 10.1038/s41598-025-98196-5

**Published:** 2025-05-02

**Authors:** Maha A.N. Gamal, Eman M.S. El-Nagar, Marwa S. Khattab, Heba M. Salem

**Affiliations:** 1 Central Laboratory for Evaluation of Veterinary Biologics (CLEVB), Agricultural Research Center (ARC), Cairo, Egypt; 2 Genetic Engineering Research Department, Veterinary Serum and Vaccine Research Institute (VSVRI), Agricultural Research Centre (ARC), 11381 Cairo, Egypt; 3 Department of Pathology, Faculty of Veterinary Medicine, Cairo University, Giza, 12211 Egypt; 4 Department of Poultry Diseases, Faculty of Veterinary Medicine, Cairo University, Giza, 12211 Egypt; 5 Department of Diseases of Birds, Rabbits, Fish & their Care & Wildlife, School of Veterinary Medicine, Badr University in Cairo (BUC), Badr City, 11892 Cairo Egypt

**Keywords:** Chicken, Ducks, Meq gene, Pathogenicity test, Turkey, UL19 gene, Cancer, Molecular biology, Diseases, Pathogenesis

## Abstract

Marek’s disease virus (MDV) is a highly contagious tumor virus that causes detrimental outbreaks in poultry. Since its initial description, the virus’s virulence and acuteness have progressively increased. During this study, we investigated suspected tumorigenic cases of MDV-1 infection among different avian species (chicken, ducks, and turkey) in various Egyptian governorates, including Al-Sharqia, Gharbia, Dakahlia, Port Said, Damietta, and Fayoum, between 2020 and 2023. A molecular study targeting the virulent oncogenic Meq gene revealed that the tumorigenic masses in chicken and duck tissues were identified as virulent MDV-1, but turkeys with cauliflower-like ovarian tumors showed negative results. The isolated MDV-1 strain of chicken origin was given the designation YLE2021 and the sequence was submitted to GenBank with accession number PQ59985. Sequence analysis revealed a partial Meq open reading frame encoding 296 amino acids and contains seven proline motifs, three of them are interrupted (187 PLQPP 191, 195 PAPP198, 224 PPQPP 228). Experimental infection of one-day-old specific-pathogen-free (SPF) chickens with a strain recovered from a chicken tumor resulted in 40% of infected birds showing the classical neural form of MDV infection. No parenchymal tumors were observed, and the virus could be molecularly detected in the peripheral blood mononuclear cells (PMNCs) of infected and neighboring uninfected SPF birds. In conclusion, this is the first report to identify the presence of MDV-1 in Egyptian ducks. Further investigations are recommended to detect the main cause of the turkeys’ tumor. Continuous molecular monitoring of circulating field viruses is crucial to investigate the mechanisms behind the increase in virus evolution, which could lead to increased virus virulence and allow the virus to evade vaccine protection.

## Introduction

Avian tumor viruses pose a significant hazard in avian production sectors, resulting in substantial global financial losses^1,2^. Marek’s Disease (MD) is a highly contagious, lymphoproliferative disease caused by a herpes virus of significant economic importance^3^. It was first described in 1907 by Josef Marek^4^, who initially observed polyneuritis in affected birds without significant mortality. However, these birds later developed tumor masses in lymphoid organs (**5).** Marek’s disease virus (MDV) belongs to the genus *Mardivirus* within the *Herpesviridae* family, sub-family *Alphaherpesvirinae*. Previously classified into three serotypes (MDV-1, MDV-2, and MDV-3, including herpesvirus of turkeys, HVT), it has been recently reclassified as *Gallid alphaherpesvirus 2* (GaHV2), *Gallid herpesvirus 3* (GaHV3), and *Meleagrid herpesvirus 1*(MeHV1)^6^.

According to Witter et al.^7^, MDV-1 have four pathotypes: mild (mMDV), virulent (vMDV), very virulent (vvMDV), and very virulent plus (vv + MDV) strains, and this virulence is recorded even in fully vaccinated flocks^8^.

According to the World Organization for Animal Health **(**WOAH)^9^MDV-1 infection presents in various forms. The classical form is primarily characterized by polyneuritis, resulting in paralysis of the wings and legs, with a loss of the normal nerve glistening appearance and cross-striations. The acute form is mainly characterized by neoplastic transformation of CD4 + T cells and lymphoma formation in visceral tissues. Peripheral nerve involvement may also occur^5^. The ocular form involves changes in one or both eyes’ iris, leading to pupil distortion. The cutaneous form is characterized by nodular lesions on feather follicles, leading to virus shedding from these follicles and subsequent contamination of the poultry house dust and dander^10,11^. Lymphoid tumors can also develop in the kidneys, spleen, lungs, and gonads^12^.

MDV encodes more than 200 genes. Marek’s *EcoRI*-Q (Meq) gene is a unique oncogene that plays a crucial role in viral pathogenesis and oncogenesis^13^. Meq is a basic leucine zipper (b-ZIP) protein, 1020 bp long encoding 339 amino acids. It shares characteristics with several viral oncoproteins, including HBZ of HTLV-I^14^, v-Jun^15^, and EBNA-3 C of Epstein-Barr virus (EBV)^16^, and Tat of HIV^17^. Meq is involved in several biological processes in chickens, including replication, immunosuppression and oncogenesis^18^. The presence of Meq polymorphisms in the proline repeats region (PRR)^19^and the b-ZIP domain within in the transactivation domain^20^has been associated with the virulence level of MDV strains^21^. In Egypt, vaccination against MDV is routinely administered in commercial hatcheries^22^, while HVT + CVI988/Rispens are included in vaccination programs for layers^23^. Despite MDV immunization, the epidemiological situation of MDV in Egypt remains poorly understood^24,25^ due to the high incidence of suspected clinical cases and tumors.

Therefore, the aim of the current work is to molecularly detect circulating virulent Marek disease virus field strain (MDV) in chicken, ducks, and turkeys in different Egyptian governorates during 2020 to 2023. This will be followed by pathogenicity testing of chicken-isolated MDV in SPF chickens and the detection of the histopathological changes in the suspected tissues.

## Materials and methods

### Ethical statement

SPF chicks were reared and handled in accordance with the guidelines and regulations the Institutional Animal Care and Use Committee (IACUC), under ethical approval number Vet CU 8/03/2022/409. All methods were performed in accordance with relevant guidelines and regulations. Bird handling was performed by experienced veterinarians, and all experimental procedures adhered to ARRIVE guideline 2.0 for the care and use of laboratory animals.

### Samples collection

Suspected visceral tumorigenic organs were collected from 2020 to 2023 from six-layer chicken flocks (Cobb and Hubbard breeds), four duck flocks (Muscovy and French breeds), and one turkey flock that were admitted to a private poultry clinic as freshly dead birds for postmortem (PM) examination for preliminary diagnosis. Only three layers flocks, consisting of one-day-old chicks, received a 0.2 ml subcutaneous (s.c.) HVT vaccine (1500 PFU/dose). The other duck and turkey flocks were not vaccinated.

From the suspected investigated flocks, approximately 100 samples (78 chickens, 18 ducks and 4 turkeys) were collected from different Egyptian governorates, including Al-Sharqia, Gharbia, Dakahlia, Port Said, Damietta, and Fayoum. The most neighboring duck and chicken flocks were in Dakahlia and Damietta.

The PM examination of the suspected chickens and ducks revealed diffuse, nodular enlargements in internal organs, including liver, spleen, pancreas, ovary, oviduct, testicles, intestine, kidney and heart. In contrast, the PM examination of the turkey samples revealed a typical cauliflower appearance of the ovaries.

All tumor-associated organs were preserved in 10% neutral buffered formalin for histopathological examination. Additionally, another portion of each tumor was sectioned and preserved at − 20 °C for DNA extraction and molecular analysis.

### Histopathological findings from cut sectioned suspected organs

Tissue specimens from tumors were collected and fixed in 10% neutral buffered formalin. Tissues were then processed using the paraffin embedding technique, sectioned into 4-µm thick sections, deparaffinized, and stained with hematoxylin and eosin.

### Genomic DNA extraction

Before proceeding with molecular identification of MDV-1, differential molecular diagnosis of the tumor tissue was performed to exclude other commonly spread tumor viruses, especially avian leukosis subgroup J virus. Thus, RNA was extracted, and RT- PCR was performed according to Soliman et al.^1^.

Tumorigenic tissues were mechanically homogenized by PRO 200 homogenizer (Pro Scientific, USA). DNA was extracted using standard procedures. Homogenized tissues were lysed in lysis buffer, extracted twice with phenol-chloroform-isoamyl alcohol (25:24:1), followed by DNA precipitation with absolute isopropanol, washed with 70% ethanol, then dried at room temperature, and finally resuspended in nuclease-free water. The extracted DNA was preserved at − 20 °C till use^26^.

### Screening of Marek’s disease virus (MDV-1) from tumorigenic tissues through amplification of the major capsid protein (UL19 gene)

The presence of MDV-1 in the tumorigenic organs was molecularly confirmed by amplification of the UL19 gene. A set of primers was designed to target the UL19 gene of MDV-1 (Table 1). The UL19 gene was amplified using 2X Easy Taq PCR Supermix (Cat. No. 011105) according to the manufacturer’s instructions. The amplification thermal cycling conditions were as follows: initial denaturation at 94 °C for 5 min, followed by 35 amplification cycles were done as follows: annealing at 55 °C for 30 s, and extension at 72 °C for 1 min.).

**Table 1:** Primers for MDV screening and the oncogenic Meq gene.

Gene	Sequence	Product size	Position	References
**UL19**	F 5΄-CCC GAT ATT ATC ATT TCA CC-3΄ R 5΄-CTC GCA TTA TTA TCT GAA GT-3	521 bp Annealing 52 °C	48,012 -> 48,031 48,532 -> 48,513	^48^
**Meq**	F: 5’-GGCACGGTACAGGTGTAAAGAG-3’ R: 5’-GCATAGACGATGTGCTGCTGAG-3’	1081 bp Annealing 60.2 °C	134,727 -> 134,748 135,984 -> 135,963	^49^

The PCR product was then electrophoresed on a 1% agarose gel, stained with ethidium bromide, and visualized under a UV transilluminator. A100 bp DNA ladder (GenRuler DNA ladder, Thermo Fisher Scientific, Cat. No. SM0314) was used to determine the size of the amplicons.

### PCR amplification of MDV-1 virulent-associated gene (Meq gene)

Only samples from chickens and ducks that showed positive PCR results for the UL19 gene of MDV-1 in the previous step were used for Meq gene amplification. Sets of primers targeting the Meq gene were designed using the Laser-gene molecular biology suite V15 (Table 1). A 1-Kb DNA Ladder RTU (GeneDirex, Cat. No. DM010-R500) used to determine the size of the amplicons.

### Meq gene sequencing

The PCR product of the amplified Meq gene was loaded onto a 1% low-melting agarose gel, sliced, and purified using the Qiaquick Gel Extraction Kit (Cat. No.28704). Sequencing was performed by GATC Company, Germany, using an ABI 3730xl DNA sequencer. Nucleotide sequence analyses were conducted using the LaserGene sequence analysis software package (LaserGene, version 10; DNASTAR, Inc.). Alignments were carried out using the Clustal W module with GenBank records (Table 2). The MDV-Meq gene of chicken origin is named YLE2021 and submitted to GenBank with accession number PQ59985.

**Table 2:** MDV GenBank records used for bioinformatics analysis of the Meq gene of strain YLE 2021 and construction of a phylogenetic tree.

Strain	virulence	country	PPPP repeats	Accession no
**CVI988**	Attenuated	Commercial vaccine	7	DQ534538
**L**	Very virulent plus	USA	2	AY362717.1
**Egypt_5**	n/a	EGYPT	4	KC161221.1
**CU-2**	Mild	USA	7	AY362708.1
**Md5**	Very virulent	USA	4	AF243438.1
**KeralaMty-2 F**	n/a	India	5	MK584541
**N/A**	n/a	India	5	MK5845443
**G2**	**Very virulent**	**China**	**5**	**AF493556.1**
**EL-Sharqyia 2015**	Very virulent	Egypt	5	MF773445
**Kafr-Elsheikh 1–2015**	Very virulent	Egypt	5	MG913293
**EL-Dakahlia 1–2016**	Very virulent	Egypt	5	MG913294
**Domiate 1–2015**	Very virulent	Egypt	5	MG913296
**EL-Gharbia 1–2016**	Very virulent	Egypt	5	MG913297
**Egypt1**	n/a	Egypt	3	JX467678.1
**Egypt 2**	n/a	Egypt	3	JX467679.1
**Egypt 3**	n/a	Egypt	3	JX467680.1
**Egypt 4**	n/a	Egypt	3	KC161220.1
**Egypt 5**	n/a	Egypt	3	KC161221.1
**HNGS201**	n/a	China	7	HF546085.1
**WS03**	Very virulent	China	3	HQ638152.1
**n/a**	Very virulent	China	3	MN94325
**Strain YLE 2021**	**Very virulent**	**Egypt**	**7**	PQ59985

n/a: not available.

### Experimental infection of SPF chickens with Meq- positive MDV-1 homogenate of chicken origin

**Fig. 1:**
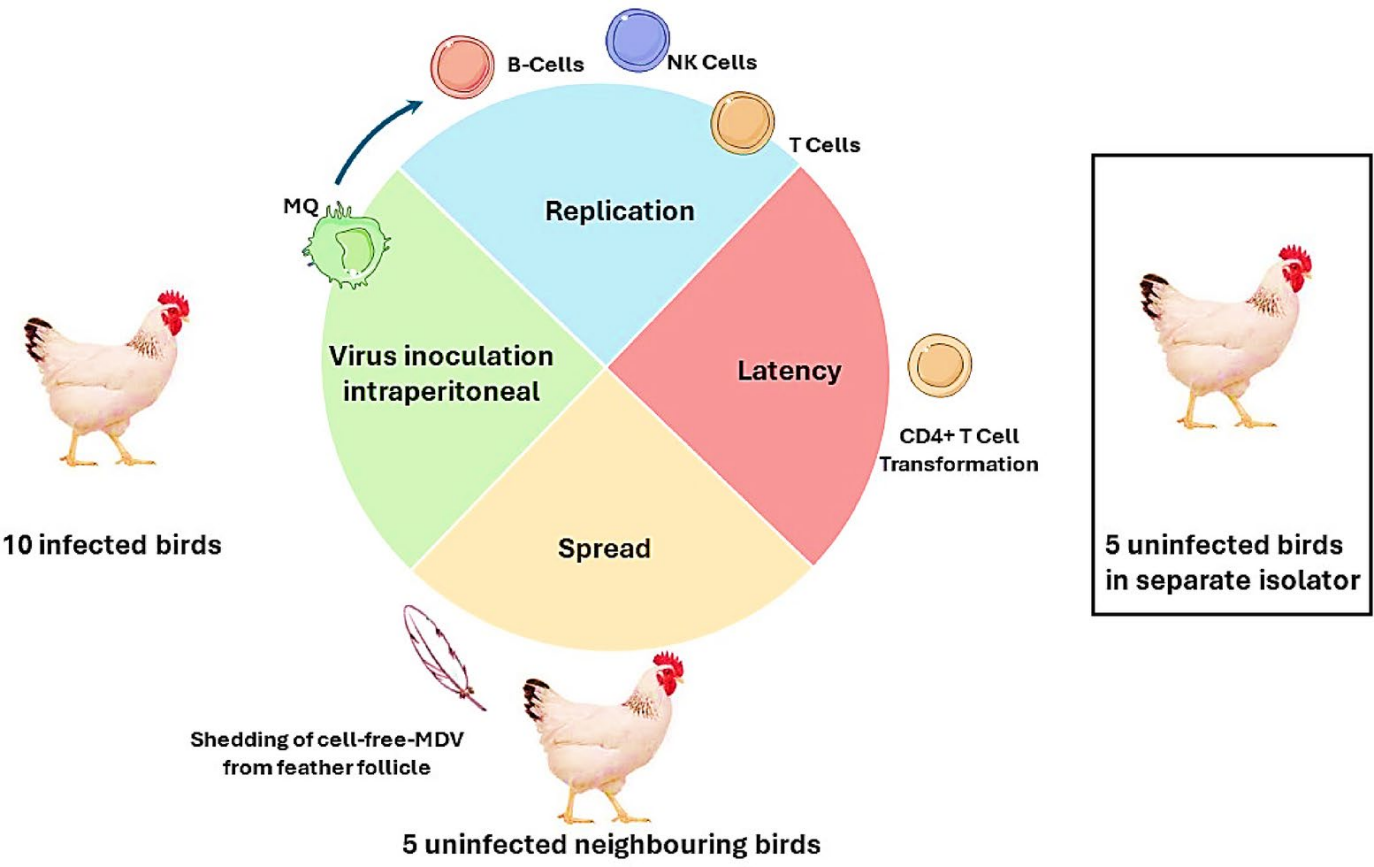
Experimental design of SPF chicken infected with field MDV-1.

Due to the unavailability of SPF ducks and turkeys in Egypt, only chicken-isolated MDV was tested for its pathogenicity in SPF chickens. Twenty-one-day-old SPF chickens (obtained from the national SPF poultry project at Koom Oshim, Fayoum, Egypt) were used in this experiment. Ten birds were inoculated intraperitoneal with 0.5 ml of liver homogenate from a positive chicken sample. Another five birds were kept uninfected and reared in a neighboring isolator to study virus spread, while the other five birds were kept in a separate isolator and remained uninfected **(**Fig. 1**)**. Chickens were kept in the isolators of Veterinary Serum and Vaccine Research Institute (VSVRI). During the experimental period (3 months), birds were continuously supplied with autoclaved ration and water *ad libitum*and maintained under adjusted light, temperature, and humidity according to the birds’ requirements, following NRC^27^. The chickens were observed for three months. Blood samples were collected weekly from the wing vein to detect the presence of the virus in peripheral mononuclear cells (PMNCs) using PCR targeting the Meq gene. Illness, lameness, wing paralysis, and eye and leg lesions were recorded. Additionally, histopathological examination of the sciatic nerve was performed.

## Results

### Gross lesions in field clinical cases

**Fig. 2:**
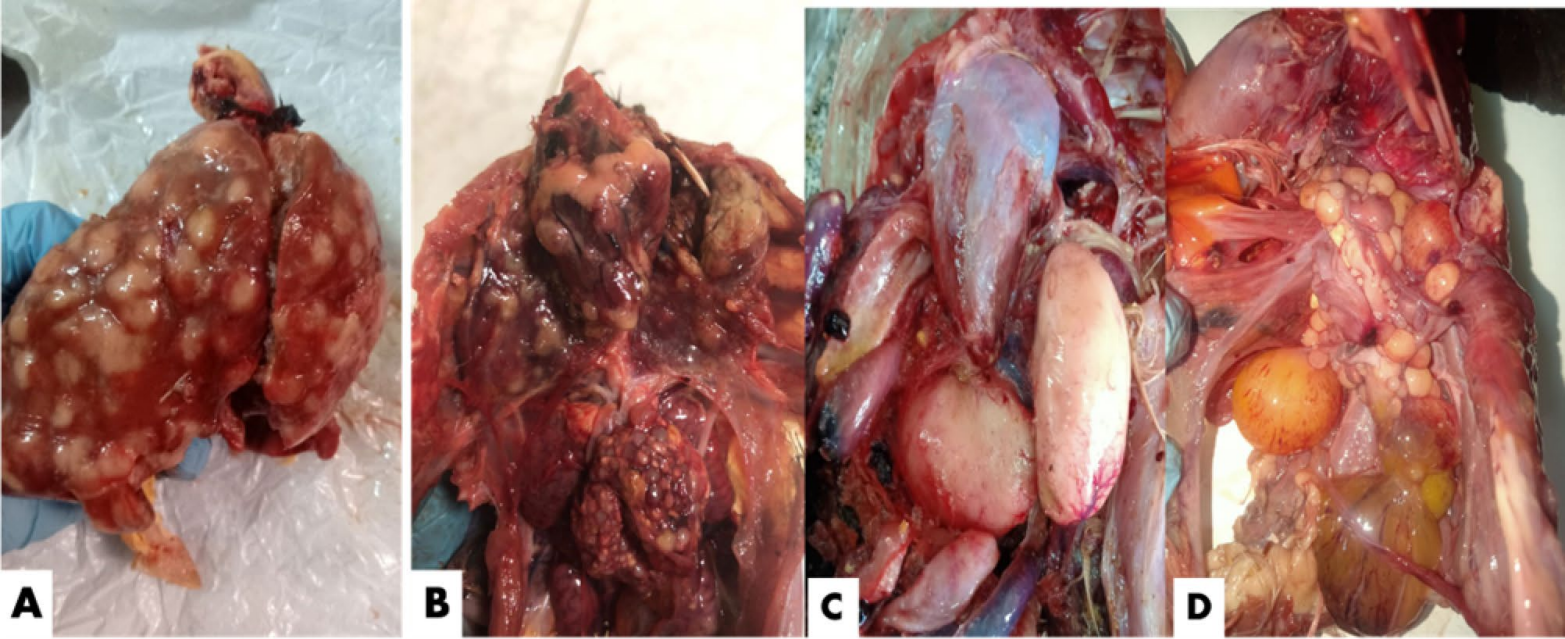
Postmortem examination findings in freshly deceased chickens suspected of being infected with Marek’s field strain showing (**A**) Nodular tumor in the liver; (**B**) Nodular tumor on the heart, lung, and ovary; (**C**) Diffuse enlargement of the testicles; (**D**) Misshaped ovary with a tumor mass.

**Fig. 3:**
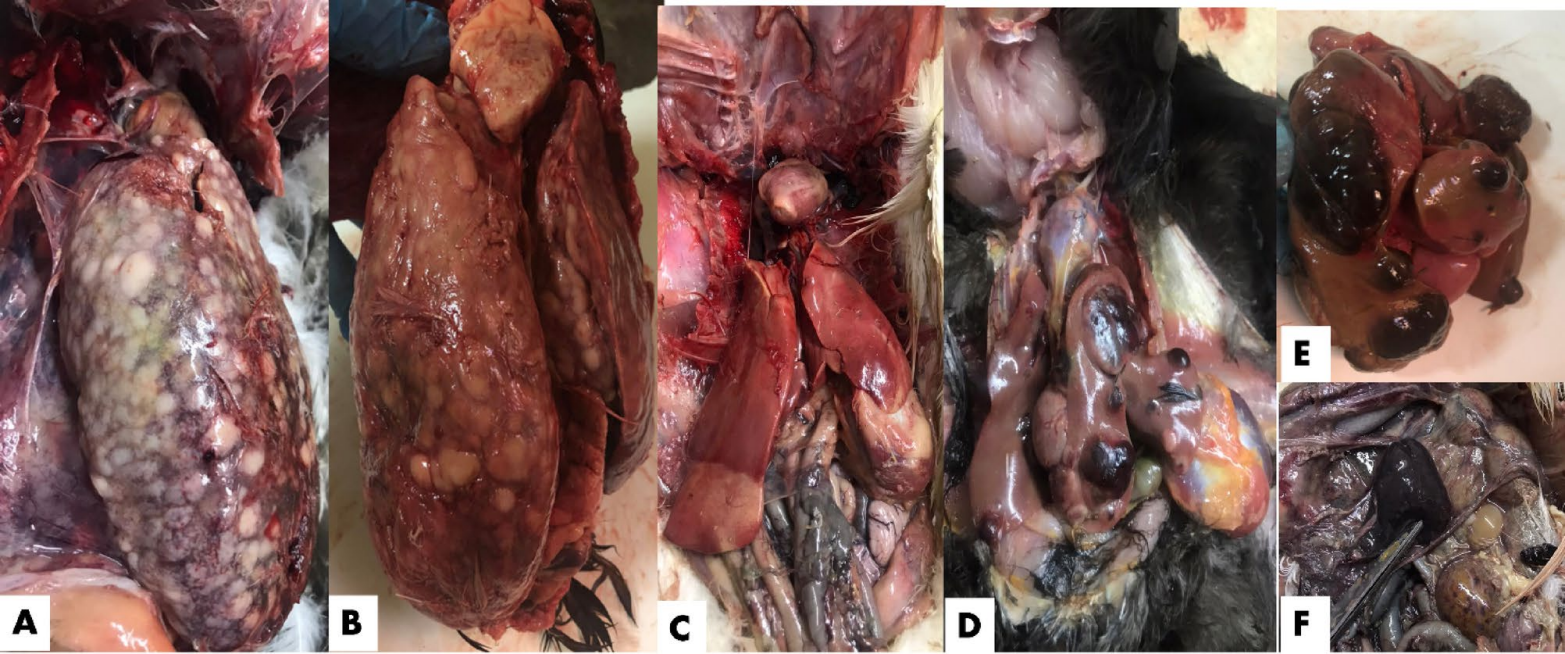
Postmortem examination findings in freshly deceased ducks suspected of being infected with Marek’s field strain showing (**A**, **B**) Nodular tumor in the liver; (**C**) Diffuse enlargement in hepatic loop; (**D** & **E**) Black nodular tumor in the liver; (**F**) Diffuse enlargement of the spleen.

**Fig. 4:**
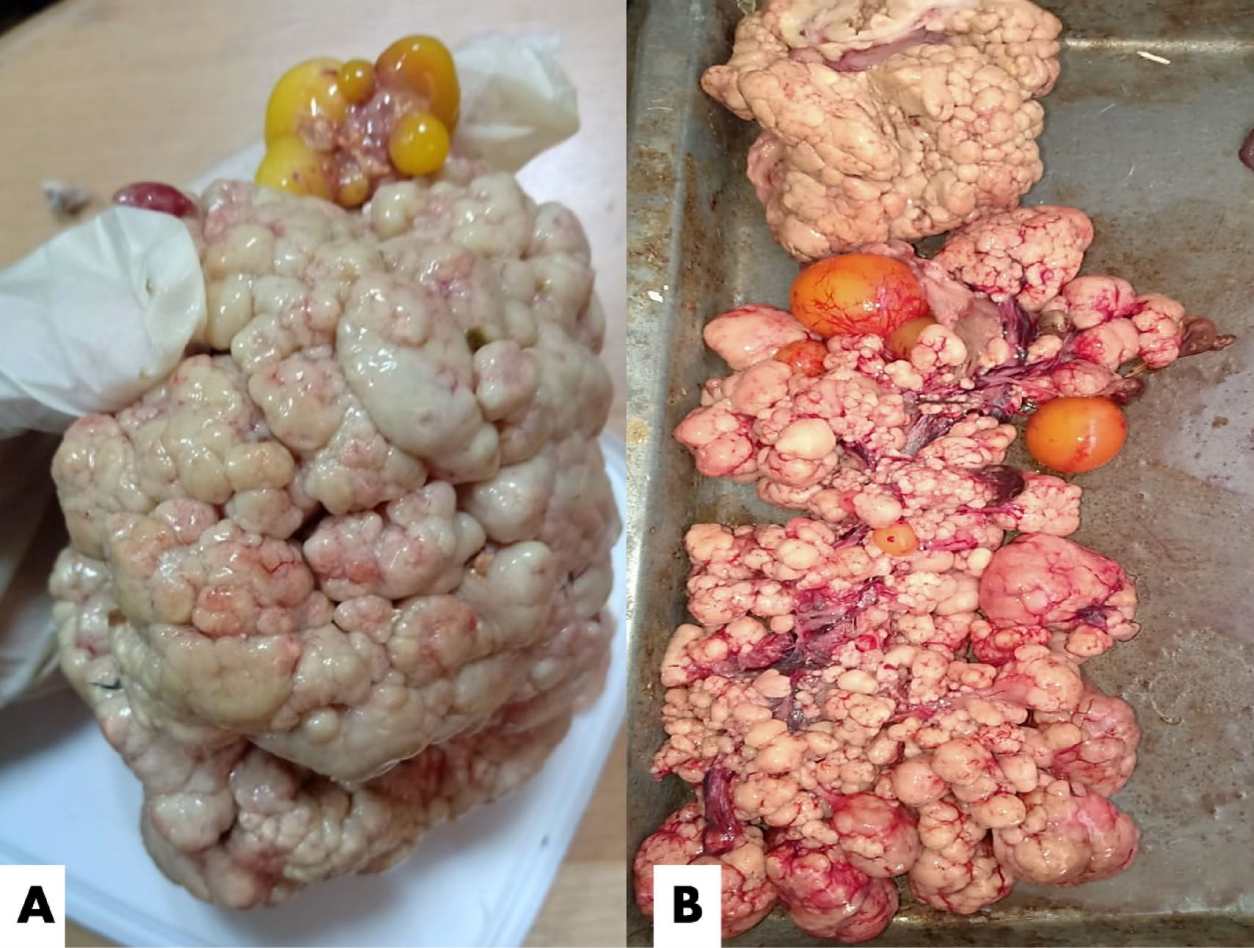
Postmortem examination of a freshly deceased turkey suspected of being infected with Marek’s disease infection showing (**A & B)** Cauliflower-like appearance of the ovary.

As seen in Fig. 2, during the clinical examination of chicken samples, livers were enlarged and showed multiple tumors-like nodules of variable sizes. Some livers exhibited caseation upon cut section, while in a few cases, the liver displayed a very soft, fragile texture. The spleen was significantly enlarged and congested. In a single chicken, a tumor was observed in the heart, a finding reported for the first time in this study. In a few cases, nodular tumors were observed in the lungs. Kidneys were also severely enlarged and extremely hemorrhagic. In only one farm, diffuse enlargement of the testicles was observed, although microscopic examination of the testes revealed normal histological structure. On the other hand, duck livers demonstrated enlargement with a greenish tint. Some livers exhibited yellowish or blackish nodules on the liver surface **(**Fig. 3**)**. Turkey cases displayed typical cauliflower-like appearance in the ovaries **(**Fig. 4**)**.

### Histopathological findings from cut sectioned suspected organs of the clinical cases

**Fig. 5:**
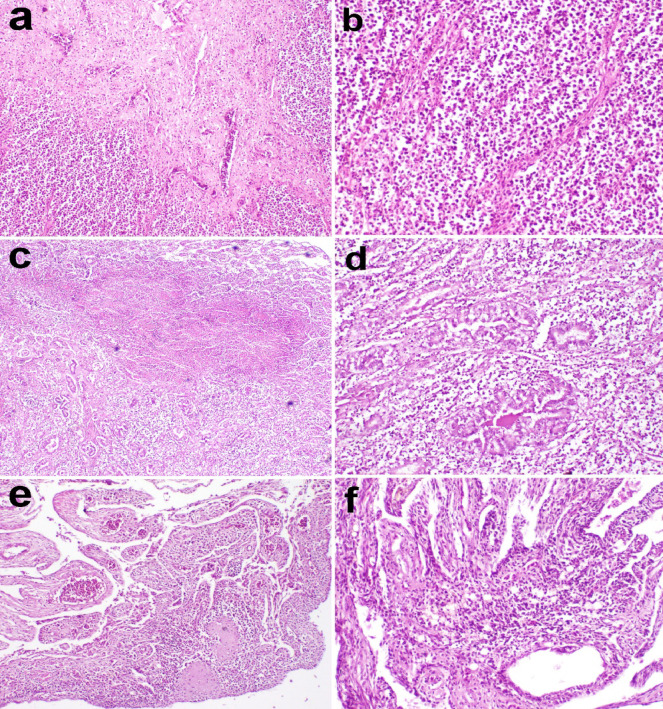
Micrographs of organs from clinical cases of ducks with suspected Marek’s disease infection. (**a**) Liver showing periportal fibrosis and severe infiltration of pleomorphic leukocytic cells, replacing the normal hepatic parenchyma (X100). (**b**) Pleomorphic cells, including small and medium-sized lymphocytes and lymphoblasts, formed multiple foci separated by fine stroma in the liver (X200). (**c**) Kidney exhibiting severe infiltration of pleomorphic cells in the peritubular interstitial tissue and a large focal area of necrosis (X40). (**d**) Peritubular infiltration of pleomorphic cells in the kidney (X200). (**e**) Ovary showing infiltration of pleomorphic leukocytic cells associated with atretic oocytes (X40). (**f**) Pleomorphic leukocytic cells infiltrating the interstitial tissue of the ovary (X200). Hematoxylin and eosin stain.

In clinical cases of ducks with suspected Marek’s disease infection, microscopic examination of the liver revealed periportal fibrosis and severe infiltration of pleomorphic leukocytic cells, replacing the normal hepatic parenchyma. The pleomorphic cells were comprised of small and medium-sized lymphocytes, as well as lymphoblasts, which formed multiple foci separated by fine stroma in liver **(**Fig. 5a, b**).** Microscopic examination of the kidney showed severe infiltration of pleomorphic cells in the peritubular interstitial tissue and large focal area of necrosis **(**Fig. 5c, d**)**. The ovaries exhibited infiltration of pleomorphic leukocytic cells associated with atretic oocytes **(**Fig. 5e, f**).**

**Fig. 6:**
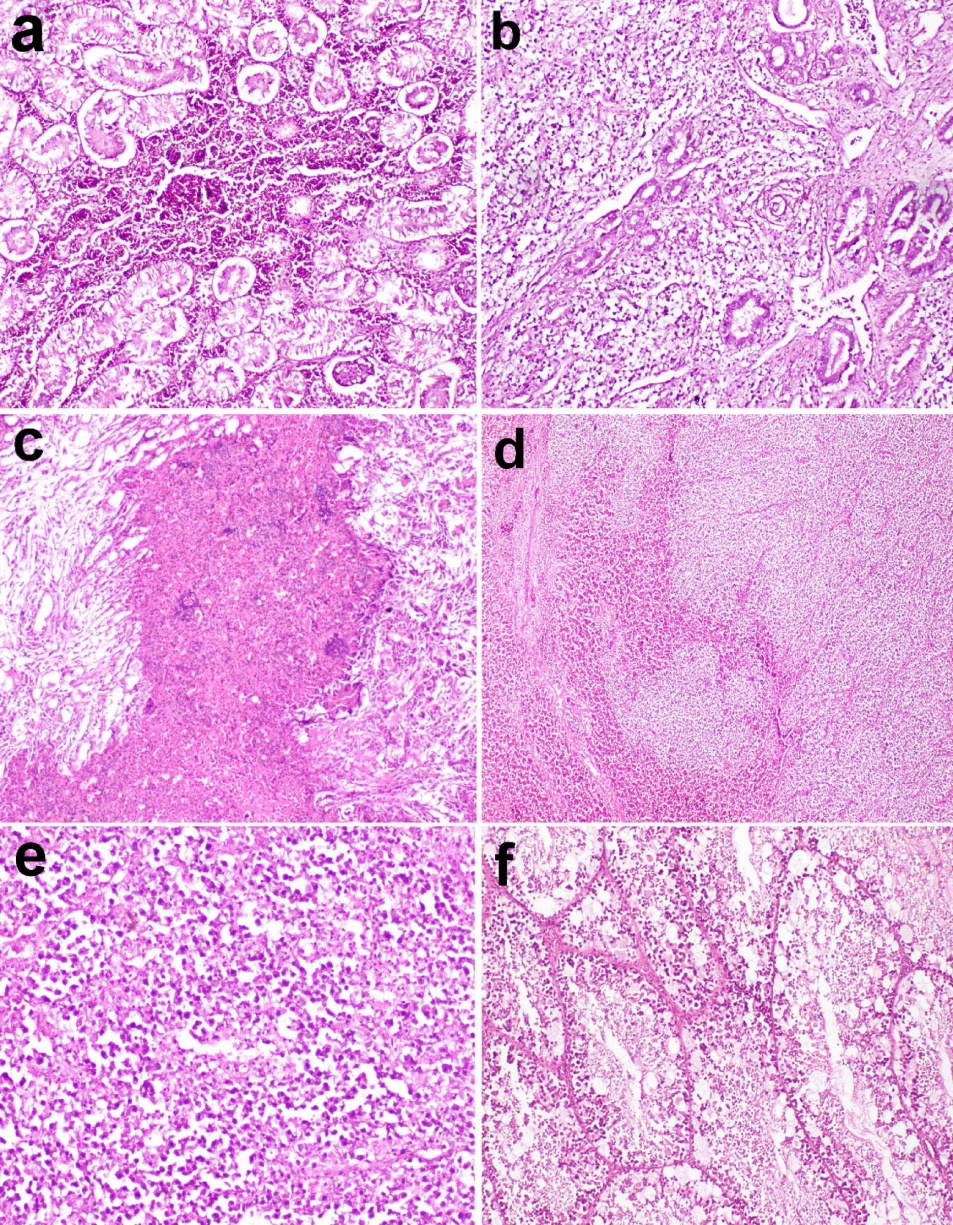
Micrographs of organs from clinical cases of chickens. Kidney sections showing (**a**) areas of hemorrhage (X 200) and (**b**) areas of leukocyte cells infiltration (X 200) (**c**) areas of caseation (X100) (**d** & **e**): The liver and spleen histopathology revealed areas of focal leukocyte infiltration masking the normal of the organ architecture. (liver: X40, spleen: X200). (**f**): Normal histological structure of testis (X200). H and E stain.

Microscopic examination of the kidney in chickens revealed areas of hemorrhage, necrobiotic changes of renal tubules, and areas of leukocyte cells infiltration, in addition to areas of caseation **(**Fig. 6**)**. Histopathological examination of the liver and spleen revealed areas of focal leukocyte infiltration, masking the normal architecture of the organs **(**Fig. 6**)**. However, microscopic examination of the testis showed normal histological structure **(**Fig. 6**)**.

**Fig. 7:**
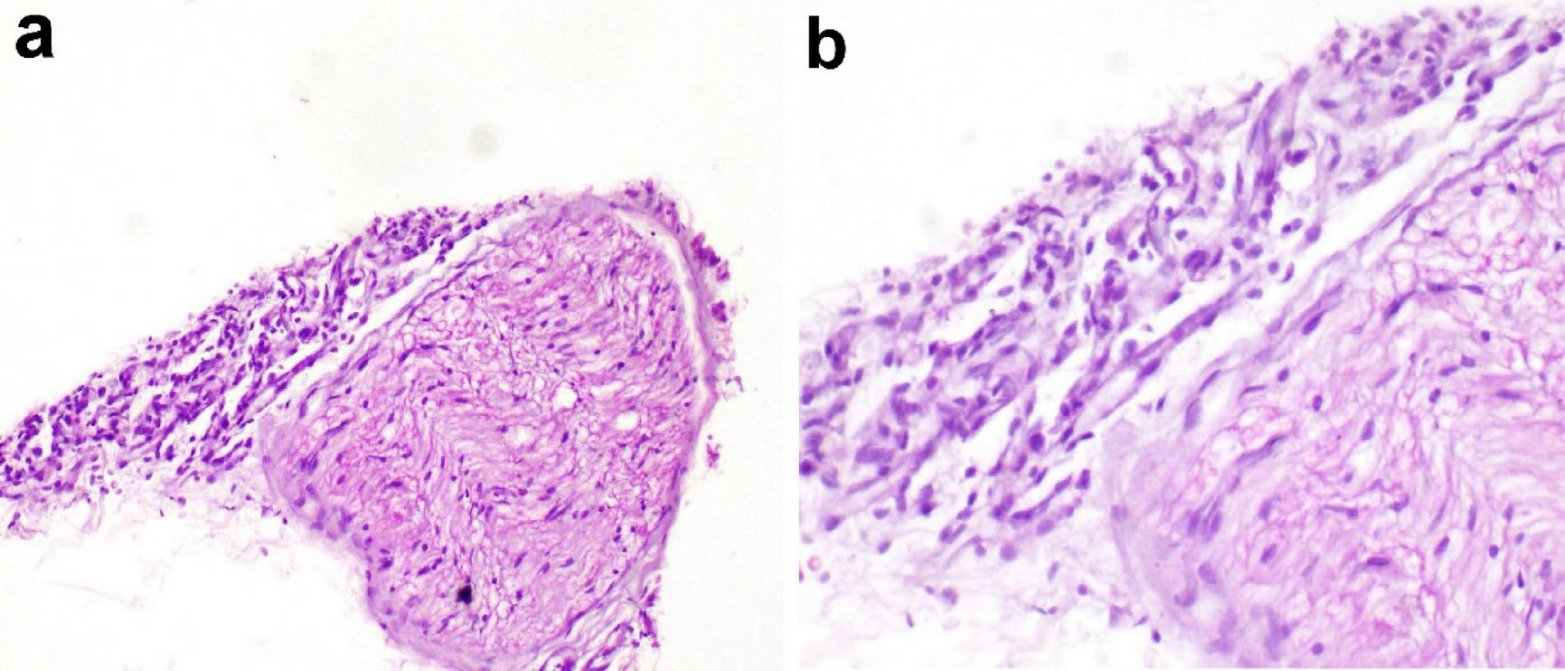
Micrographs of nerve of chickens experimentally infected with Marek’s disease (MDV) showing severe perineuronal mononuclear leukocyte infiltration and few leukocytes between nerve fibers (H and E stain).

Microscopic examination of nerves in chickens experimentally infected with MD revealed demyelination of nerve fibers, infiltration of a few mononuclear leukocytes into the nerve fibers, perineuronal edema, and numerous leukocytes surrounding nerve fibers. Perivascular mononuclear leukocytes were also observed **(**Fig. 7).

### Detection of MDV-1 from tumorigenic organs through amplification of the UL19 gene

**Fig. 8:**
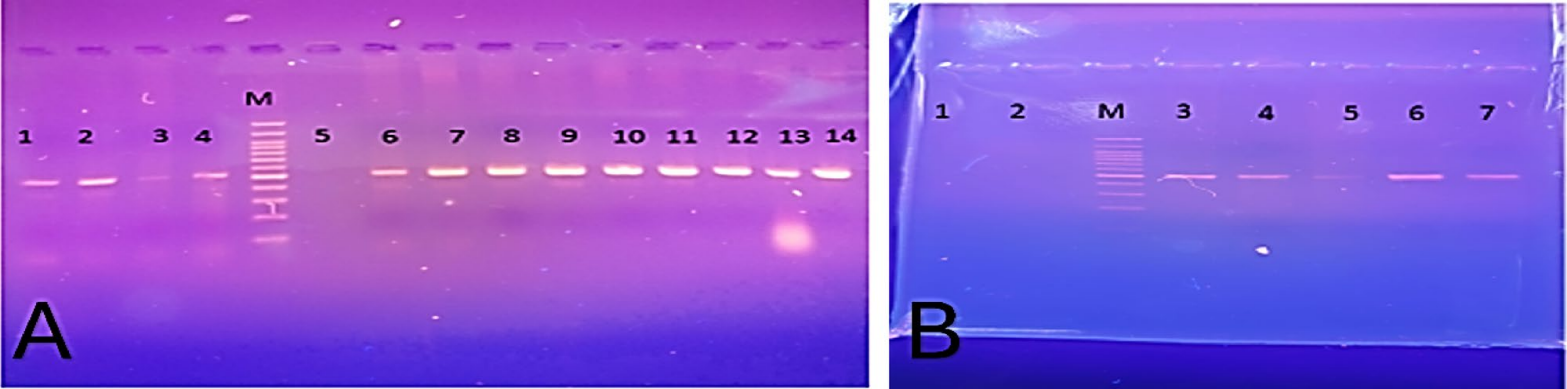
(**A)** Representative PCR amplification showed clear positive bands at 500 bp corresponding to the UL19 gene. Lanes 1–4: positive MDV duck origin; lane 5: negative turkey-associated tumor; lanes 6–14: MDV chicken origin. M: DNA ruler 100 bp plus marker. (**B)** Representative PCR amplification showed clear positive bands at 500 bp corresponding to the UL19 gene. Lane 1: negative turkey-associated tumor; lane 2: negative control (Tris); lanes 3,4: positive MDV duck origin; lanes 5,6: MDV chicken origin; lane 7: control positive vaccine (Live attenuated T. C. Marek Rispens). M: DNA ruler 100 bp plus marker.

All tumor tissues were molecularly negative for the avian leukosis virus. Regarding molecular identification of MDV-1, out of 78 chicken samples, only 33 were positive (42.3%). The distribution of positive samples was as follows: 12 livers (36.3%), 10 spleens (30.3%), 1 pancreas, 2 liver nodules, 1 oviduct, 1 heart, 2 lungs, and 4 kidneys. Meanwhile, out of 18 duck samples, 9 were positive (50%), while all turkey tumor samples were negative **(**Fig. 8A&B).

### PCR amplification of the MDV-1 virulence-associated gene (Meq gene)

**Fig. 9:**
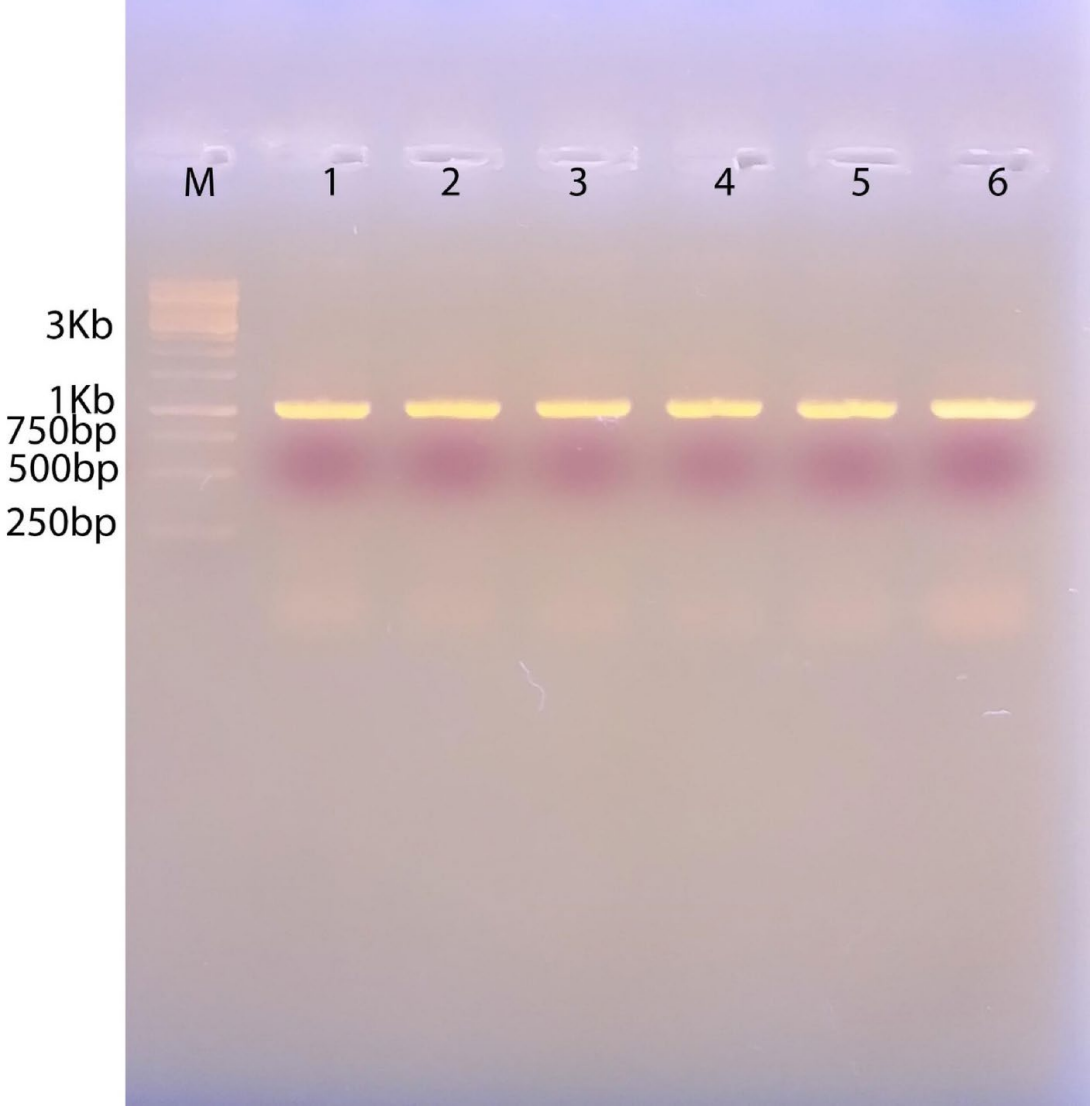
Representative PCR amplification of MDV from chicken, duck tumor, and PMNCs, showing positive amplification of the virulent tumorigenic Meq gene. Clear bands at 1000 bp correspond to the target primer. M: DNA marker, 1 Kb DNA ladder RTU, Lanes 1–6: positive samples at 1000 bp. Lane 1, 2: duck tumor, lane 3: chicken tumor, lane 4: PMNCs from infected chickens, lane 5: PMNCs from neighboring chickens, lane 6: control positive CVI988/Rispens).

All positive MDV tissue samples, along with the isolated PMNCs from the experimentally infected chickens (15 days post-infection and after one month from uninfected neighboring chicks), were molecularly positive when tested with the specific primer targeting the oncogenic Meq gene (Fig. 9). All samples showed a 1086 bp product corresponding to the amplification target **(**Fig. 8).

### Sequencing analysis of Meq gene

**Fig. 10:**
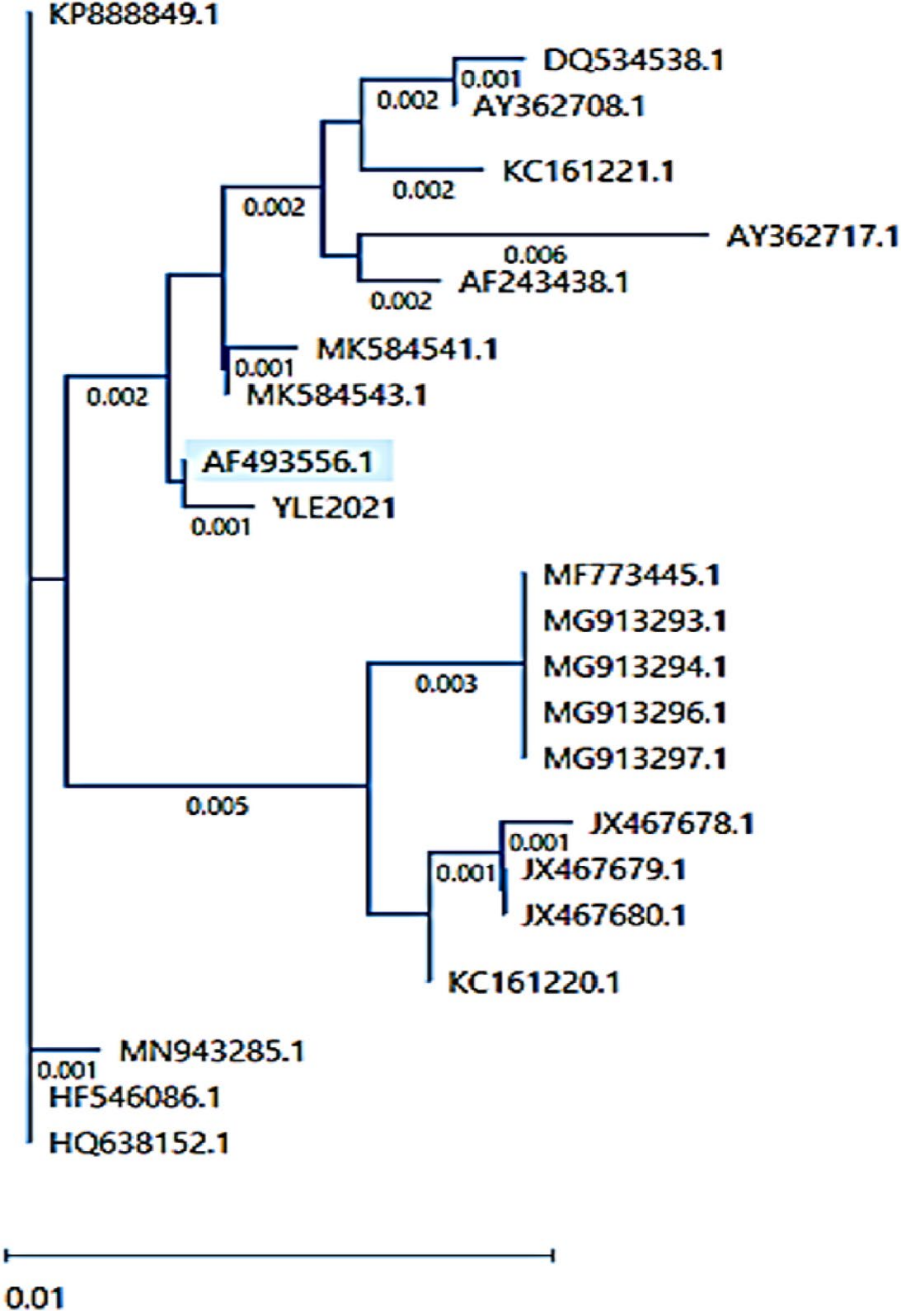
Phylogenetic analysis of MDV based on nucleotide sequence of the Meq gene using a distance-based neighbor-joining method. The strain in this study is named YLE2021. The blue strains refer to the very virulent Chinese MDV.

**Fig. 11:**
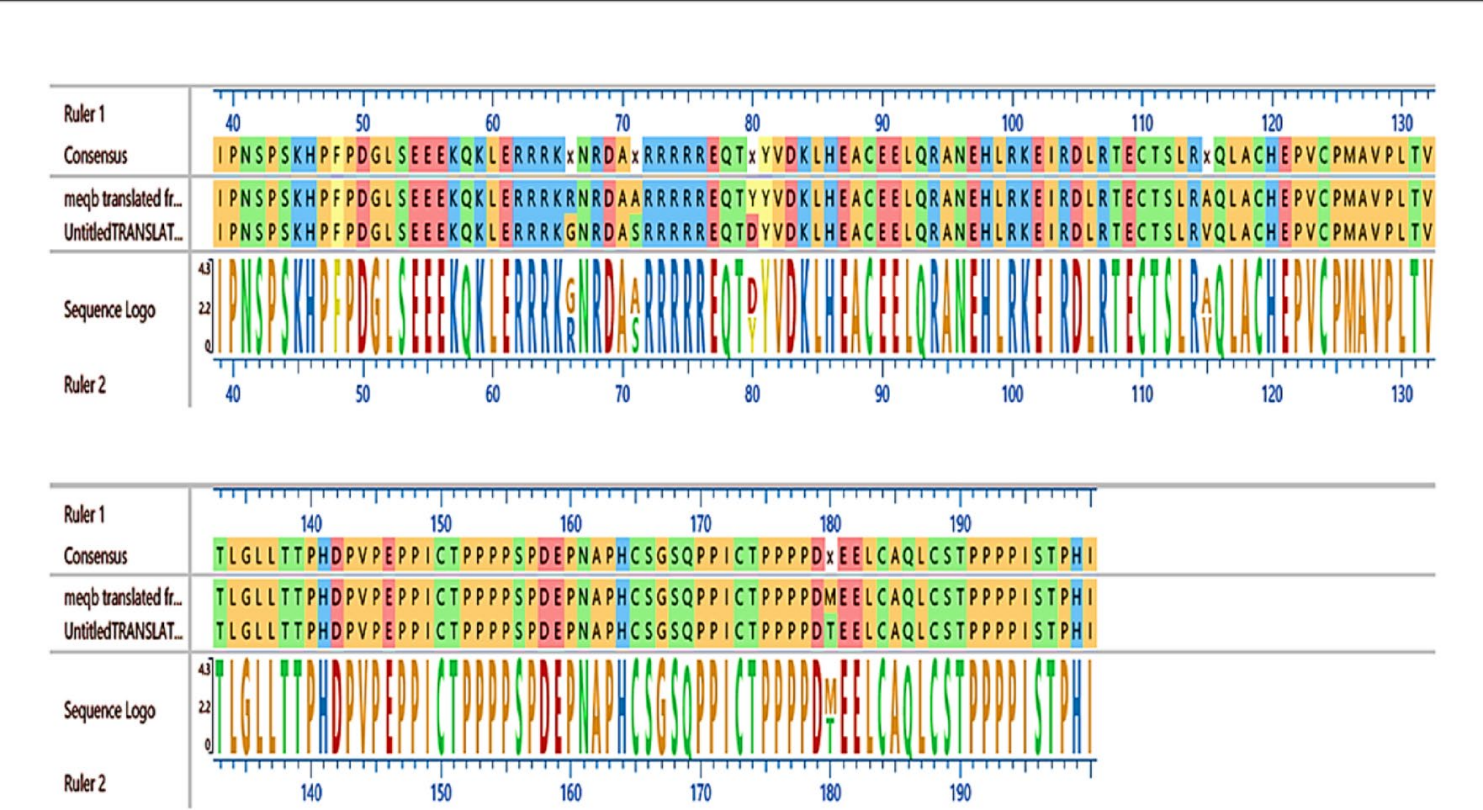
Amino acid sequence alignment of the MDV Meq in the current study (upper) with live attenuated CVI988 vaccine (lower). Positions 66, 71 of the Basic region, 80, 115 of the Leucine zipper, and 180 of the Transactivation Domain are highlighted.

As seen in Figs. 10 and 11, the identity percentages were found to be 99.98% with strain AF493556 isolated from China, 99.78% with MK5845443 Indian virus, and 99.66% with Chinese HF546086 strain. Phylogenetic analysis based on nucleotide sequences revealed that the YLE 2021 Egyptian strain, with accession number PQ59985, is closely related to the Chinese strains. Alignment between our strain and the live attenuated CVI988 vaccine revealed several differences at the amino acid level, indicating that our isolate is not a vaccine strain. The amino acid sequence of the YLE2021 Meq gene comprises 296 residues; however, this represents a partial open reading frame lacking both start and stop codons. Consequently, while the sequence provides molecular characterization and highlights differences in proline motifs (seven proline motifs, three of which are interrupted: 187 PLQPP 191, 195 PAPP 198, 224 PPQPP 228), it cannot alone be used to definitively classify the isoform or attribute virulence. Phenotypic and experimental evidence remain the primary support for the strain’s pathogenicity.

### Experimental infection of SPF chickens with Meq- positive MDV-1

**Fig. 12:**
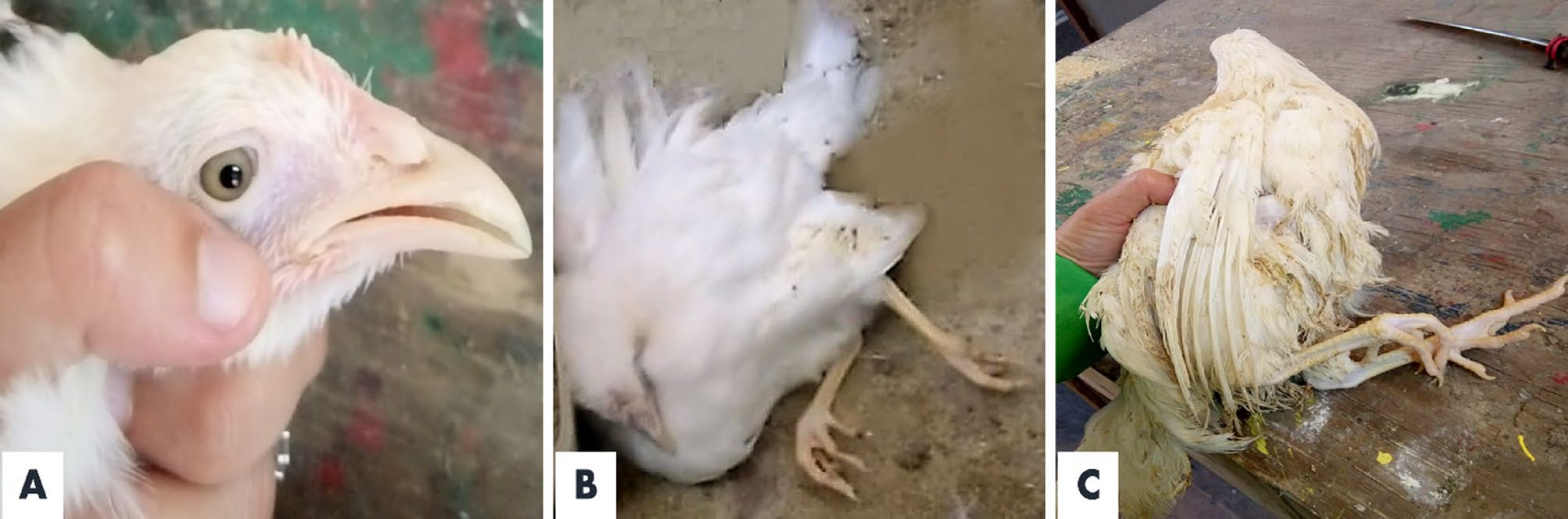
Clinical signs of experimentally infected SPF chickens with Marek’s disease virus field strain showing: (**A**) Change in iris color due to lymphocyte infiltration of the iris; (**B** & **C**) unilateral lameness, dropped wing, and recumbency with curled toes.

Experimentally infected chickens appeared depressed, with loss of appetite and weight loss and after the first month of infection, while the neighboring uninfected chicks were apparently healthy. After three months, four chickens from the infected group (40%) showed unilateral lameness, dropped wings, recumbency with curled toes and white-greyish irises **(**Fig. 12**)**. No mortality was recorded for either the inoculated or the contact groups throughout the experimental duration. PMNCs isolated from all ten inoculated and the five in-contact birds were positive by PCR targeting the Meq gene **(**Fig. 9**).**

**Fig. 13:**
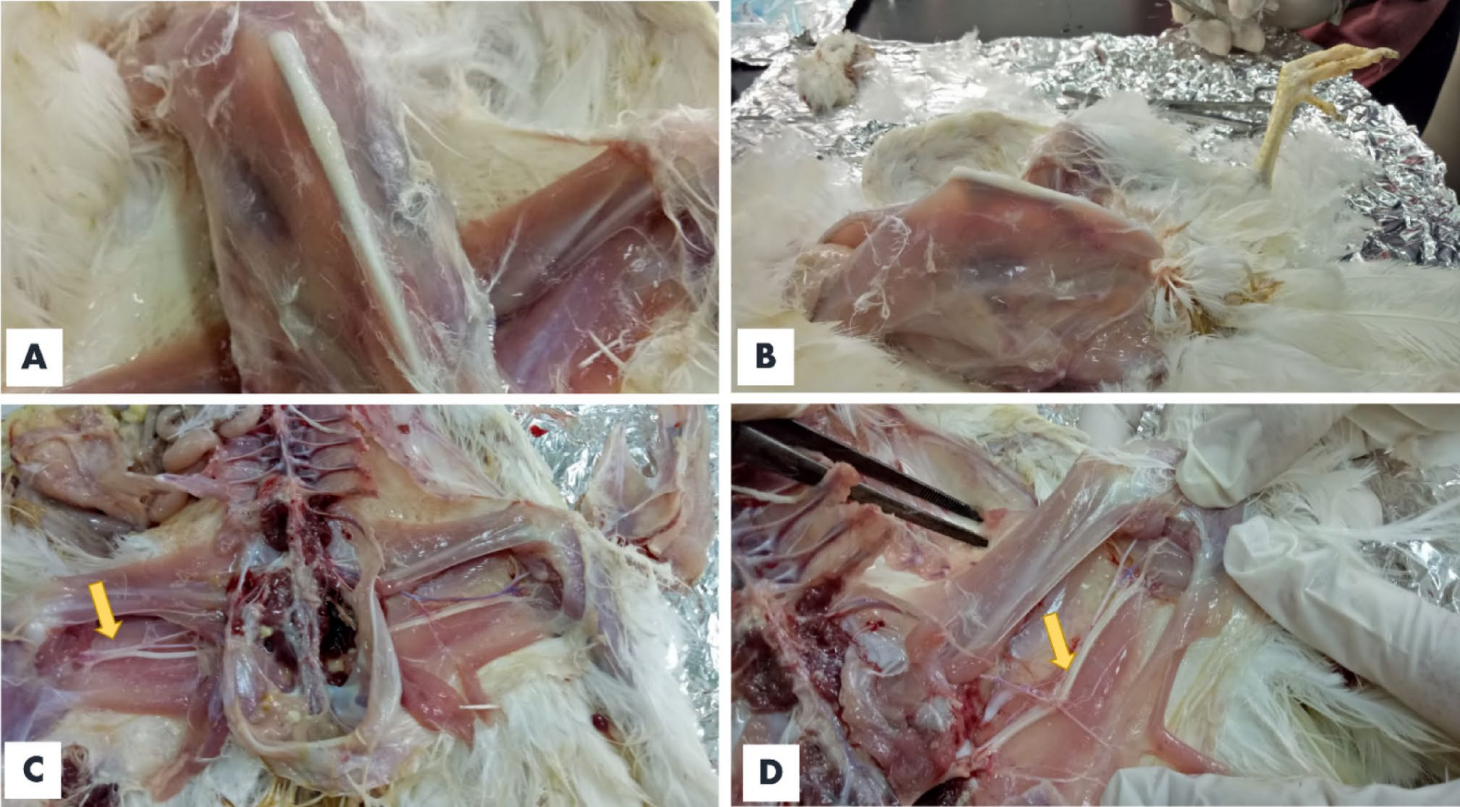
Postmortem examination of experimentally infected SPF chickens with a field strain of Marek’s disease showing (**A & B)** Severe emaciation with a protruded keel bone; (**C &D)** Enlarged, thickened sciatic nerve with loss of cross-striations (yellow arrow).

The PM examination of experimental SPF chickens at the end of the experiment showed weight loss associated with a protruded keel bone **(**Figs. 12 and 13**).** Hemorrhages were present on the liver surface, and no tumor lesion were recorded macroscopically in any parenchymatous organ. Thickening of the sciatic nerve with loss of its cross-striations was observed **(**Fig. 13**)**. On the other hand, no PM lesions were detected in the neighboring birds.

## Discussion

MDV is one of the most important and widely distributed immunosuppressive and oncogenic viruses. The disease causes a decrease in poultry productivity, as well as significant morbidity and mortality levels, resulting in major losses in the poultry sector globally^2^. MDV mostly infects domestic chickens, commonly sparrow hawks, domestic geese, pheasants, and mallards and rarely occurs in turkeys, quails, and owls^28^. Although the worldwide use of live attenuated vaccines, either monovalent (HVT) or bivalent (HVT and SB-1) and HVT + CVI988/Rispens, could dramatically reduce the losses due to MDV^29^, outbreaks and tumor formation are still increasing, and vaccinated chickens continue to shed the virus in the environment^30^. During MDV evolution, specific mutations in the genome have been reported that could be attributed to the increase in virus virulence and allow the virus to evade vaccine protection^19,31^.

Identification of a tumorigenic proliferative mass is a highly indicative sign during the diagnosis of MD; this lymphomatous mass usually consists of heterogeneous populations of small- and medium-sized pleomorphic lymphocytes and blast cells^32^. Identification of MDV infection will depend mainly on the gross appearance of visceral lymphomatous lesions along with molecular identification^9^. Molecular detection is a very helpful tool in considering whether the circulating strain is vaccinal or virulent, or even in determining the virulence degree of the virus based on the detection of pp38, vIL-8, and Meq virulent genes^33^.

During our clinic investigation, diffuse nodular tumors were observed in different visceral organs of chicken, ducks, and turkey from different localities. The tumorigenic cases in duck and chicken flocks located in Dakahlia, and Damietta were in close proximity to each other. Molecular identification using the UL19 (the major capsid protein) of MDV revealed that 42.3% of chicken samples were positive for MDV (36.3% of the positive samples were from liver tissues, and 30.3% were spleen tissue), while 50% of duck livers showed positive MDV. This result aligns with the study of MDV genome copy number^34^, where higher copy numbers were found in the liver, spleen, and heart at 90 days post infection. During evaluation of the identified virus virulence, all positive MDV liver and spleen tissues from chicken and duck origin were positive when tested molecularly with the Meq virulence gene. Screening for Meq in Egypt from 2011 to 2016^13^showed that the Meq protein from circulating samples in Egypt showed identity with hypervirulent European and Chinese isolates. Since then, many reports have attempted to screen for the virulent Meq in Egypt^24^. In the present study, the amino acid sequence of the YLE2021 chicken Meq comprised 296 residues; however, this represents a partial open reading frame. The sequence analysis revealed seven proline motifs, three of which were interrupted (187 PLQPP 191, 195 PAPP 198, 224 PPQPP 228). While these molecular observations provide information about the Meq gene, they cannot alone determine isoform classification or virulence, which is supported primarily by phenotypic and experimental evidence.

Since 1954, when Soliman et al.^35^reported the first case of MD in Egypt, all recorded cases of MDV infection have been restricted to chickens^25,36^, and no records of MDV infection in Egyptian ducks exist. Consequently, Egyptian ducks do not receive MDV vaccination. However, during this study, clear nodular tumors were recorded in both chickens and ducks and were molecularly confirmed to be caused by virulent MDV with the oncogenic Meq gene. The reason behind the emergence of tumorigenic masses of MDV infection in ducks might be attributed to the horizontal transmission from chicken farms to ducks’ farms, especially considering the close proximity of the tumor lesions observed in neighboring chicken and duck farms in Dakahlia and Damietta governorates. Similar findings were recorded during the study by Damir et al.^37^., where horizontal transmission of the Marek’s disease virus from infected flamingos to chickens occurred. These findings emphasize the importance of studying the epidemiological situation of MDV alongside molecular analysis.

Regarding MDV infection in turkeys, reports are very rare^38–40^, although some cases have been confirmed in Italy^41^. Clinical infection with MD in turkeys is not well-defined, and signs are mainly restricted to lameness and stunted growth, emerging between 12 and 30 weeks of age^42,43^. MDV diagnosis in turkeys relies heavily on molecular and histopathological lesions^43^. During this study, a clear cauliflower-like tumor was observed in turkeys. However, this tumor mass was molecularly negative for MDV, and no significant histopathological changes were observed. On the other hand, Žlabravec et al.^28^ reported the presence of visceral tumors in Slovenian commercial turkey flocks, and the visceral tumor was molecularly characterized by the GaHV2 Meq region.

During experimental infection of one-day-old SPF chickens with the recovered virulent visceral form of MDV, the main clinical signs observed were ruffled feather, dropped wing, unilateral lameness, curled toes, and incoordination. Notably, no visceral tumors were recorded. These symptoms, according to WOAH^9^, are characteristic of the classical form of MDV. The appearance of the clinical signs of the classical form may be attributed to the genetic nature of the SPF chickens. This was explained by Tregaskes et al.^44^., who studied the structure of the chicken B-cell marker (*ChB6*) gene in SPF chickens and found that the *ChB6* gene controls the regulation of B-cell development and is associated with resistance to MD and regression of *Rous- sarcoma*^44^. During the study by Shaheen et al.^45^., *ChB6*was detected in 10 native breeds, including the SPF Lohmann. No reports have documented the occurrence of tumors in these ten breeds, explaining the absence of tumors in experimentally infected SPF chickens and the observation of only nerve involvement. At 15 days post-infection, the virus could be isolated from the PMNCS and from the neighboring birds after one month. These results align with the studies of^46,47^, where viral DNA could be identified 5–7 days post-infection, while virus transmission (due to dust and shedding from feathers) occurred between 12 and 14 days.

## Conclusion

The tumor under study, of chicken origin, is caused by a very virulent visceral form of Marek’s disease virus (MDV) named YLE2021. This strain is closely related to the Chinese MDV very virulent strains. This is the first record of the presence of a virulent MDV visceral form in Egyptian ducks. So, it is advisable and of top priority to conduct further molecular studies to compare the sequencing and phylogenetic analysis of the virulent Meq gene isolated from both duck and chicken origins. Additionally, the implementation of MDV vaccines in duck vaccination programs should be announced. Regarding the turkey, the observed tumor mass requires further investigation to understand the reason behind its formation. Finally, the mechanism behind the increase in virus evolution, which could lead to increased virus virulence and subsequent vaccination failure, should be investigated.

## Data Availability

All resulting data from this study are included in this manuscript. The chicken YLE2021 sequence is available on GenBank with accession number PQ59985 (https://www.ncbi.nlm.nih.gov/nuccore/PQ624839.1/). Any additional datasets related to the current study are available from the corresponding author upon reasonable request.

## Declarations

### Approval for animal experiments

SPF chicks were reared and handled in accordance with the guidelines and regulations of the Institutional Animal Care and Use Committee (IACUC) under ethical approval number Vet CU 8/03/2022/409. All methods were performed in accordance with relevant guidelines and regulations. Bird handling was performed by experienced veterinarians, and all experimental procedures adhered to ARRIVE Guideline 2.0 for the care and use of laboratory animals.
