# Author Correction: Molecular discernment and histopathological features of oncogenic Marek’s disease virus among different farmed avian species in Egypt

**DOI:** 10.1038/s41598-026-44612-3

**Published:** 2026-03-17

**Authors:** Maha A. N. Gamal, Eman M. S. El-Nagar, Marwa S. Khattab, Heba M. Salem

**Affiliations:** 1 Central Laboratory for Evaluation of Veterinary Biologics (CLEVB), Agricultural Research Center (ARC), Cairo, Egypt; 2 Genetic Engineering Research Department, Agricultural Research Centre (ARC), Veterinary Serum and Vaccine Research Institute (VSVRI), Cairo, 11381 Egypt; 3 Department of Pathology, Faculty of Veterinary Medicine, Cairo University, Giza, 12211 Egypt; 4 Department of Poultry Diseases, Faculty of Veterinary Medicine, Cairo University, Giza, 12211 Egypt; 5 Department of Diseases of Birds, Rabbits, Fish & Their Care & Wildlife, School of Veterinary Medicine, Badr University in Cairo (BUC), Badr City, 11892 Cairo Egypt

Correction to: *Scientific Reports* 10.1038/s41598-025-98196-5, published online 02 May 2025

The original Article contained an error. The Meq gene sequence (PQ59985) was incorrectly described as a short version of the Meq gene rather than as a partial open reading frame.

As a result, in the Abstract:

“The amino acid sequence of the YLE2021 chicken Meq showed a 296 amino acid length (short Meq), which is characteristic of very virulent Meq”

now reads:

“Sequence analysis revealed a partial Meq open reading frame encoding 296 amino acids”

In the Results section, the ‘Sequencing analysis of Meq gene’ subsection:

“The amino acid sequence of the YLE2021 chicken Meq showed a length of 296 amino acids (short Meq), which is characteristic of the very virulent Meq and containsseven proline motifs, three of which are interrupted motifs (187 PLQPP 191, 195 PAPP198, 224 PPQPP 228).”

now reads:

“The amino acid sequence of the YLE2021 Meq gene comprises 296 residues; however, this represents a partial open reading frame lacking both start and stop codons. Consequently, while the sequence provides molecular characterization and highlights differences in proline motifs (seven proline motifs, three of which are interrupted: 187 PLQPP 191, 195 PAPP 198, 224 PPQPP 228), it cannot alone be used to definitively classify the isoform or attribute virulence. Phenotypic and experimental evidence remain the primary support for the strain’s pathogenicity.”

In the Discussion section:

“The amino acid sequence of the YLE2021 chicken Meq demonstrated a length of 296 amino acid (short Meq), characteristic of the very virulent Meq, and contained seven proline motifs, three of which were interrupted motifs (187 PLQPP 191, 195 PAPP198, 224 PPQPP 228).”

now reads:

“In the present study, the amino acid sequence of the YLE2021 chicken Meq comprised 296 residues; however, this represents a partial open reading frame. The sequence analysis revealed seven proline motifs, three of which were interrupted (187 PLQPP 191, 195 PAPP 198, 224 PPQPP 228). While these molecular observations provide information about the Meq gene, they cannot alone determine isoform classification or virulence, which is supported primarily by phenotypic and experimental evidence.”

The original Article has been corrected.

